# Goals and Success in Sport: The Perspectives of Parents and Adolescent Girls in Kayaking

**DOI:** 10.3390/bs13070580

**Published:** 2023-07-12

**Authors:** Orr Levental, Yosi Yaffe, Dalit Lev Arey

**Affiliations:** 1 Department of Physical Education, Tel Hai Academic College, Qiryat Shemona 1220800, Israel; 2 Department of Education, Tel Hai Academic College, Qiryat Shemona 1220800, Israel; 3 School of Psychology, The Academic College of Tel Aviv Yaffo, Tel Aviv-Yafo 6818211, Israel

**Keywords:** kayaking, Israel, parents, adolescent girls, sports, success

## Abstract

Setting objectives and evaluating success are intrinsically linked to motivation in sports and behavior during training and competition. According to social cognitive theory, the perception of success is divided into “task” and ”ego”, which indicate an inner orientation versus an external perspective when analyzing an athlete’s achievements. These impressions can be influenced by various factors such as maturity level, gender, and the type of activity in which they participate. The current study compares perceptions of success among parents and adolescent girls competing in kayaking in Israel. To this end, a qualitative study was conducted with 20 in-depth interviews emphasizing goal setting and individual perceptions of success. The research findings reveal that contrary to previous studies, there is a perceptual gap between the two groups, with parents measuring success primarily in terms of enjoyment and self-fulfillment, and girls referring to perceived success in the context of achievements. It was also found that there is a discrepancy between setting goals and defining success, following experience and interactions within the training. The article discusses the reasons behind these gaps and the potential ramifications for sports activity and participation.

## 1. Introduction

Hundreds of millions of children and youth across the globe regularly engage in organized sports. Their participation in diverse sports activities is driven by a wide range of personal and social motivations, not only their own, but also those influenced by their parents, family, fans, and friends [1,2]. These motivations encompass desires to improve their appearance, lose weight, boost self-confidence, widen their social circle, engage in pleasurable activities, and enjoy competitive activity, among others [3].

A significant motivation often cited in the literature Is the desire to achieve and experience success. This motivation is crucial, as perceived success influences the engagement of these athletes, shaping their behavior and feelings towards their performance [4]. However, in the case of young athletes, many factors are also contingent on the influence and motivations of their parents. Parents play pivotal roles in choosing the sports their children participate in, providing logistical support, serving as personal role models, and setting their children’s goals as participants in the chosen sport [5,6]. The importance of perceived success as a motivation for physical activity significantly influences whether someone persists in sport, and the positive and negative consequences of this participation [7]. However, when it comes to children and adolescents who engage in sport, there are two groups involved (parents and children), and these groups do not necessarily share the same perspectives regarding success. This article aims to examine the discrepancies in the views of parents and children. To this end, a qualitative analysis of in-depth interviews with parents and their daughters, who participate in kayaking in Israel, was conducted. The objective of this study is to better understand the dynamics between parents’ and children’s perceptions of success in sports, particularly in the context of kayaking in Israel.

### Perceived Success in Sport

The topic of success in sport is quite complex. Generally, success in sport can be defined as the realization of an athlete’s goals in the context of his/her sports activities [8]. Children’s perceived success in sport is largely influenced by a multitude of factors. These may include the personal goals they set for themselves, their physical abilities, psychological resilience, the quality of coaching they receive, the support from their parents, family, and friends, and their commitment and dedication to the sport [9]. Among these, subjective ethical perceptions, usually formed at a young age and shaped by interactions with parents and coaches, also play a significant role [10]. Hence, success can take the form of winning a competition, improving one’s performance, or acquiring new skills, and can be related to deriving pleasure, realizing personal growth, or forming social ties. Only a limited number of research studies have examined success in sport, which is usually defined in relation to specific activities, such as strength training [11] or general physical activity [12]. Nevertheless, the prevailing assumption is that success in sport is measured primarily by achievements. Despite the natural ambition of most athletes, only a few attain the highest levels in their sport. Yet, if achievements were the absolute measure, success would be virtually unattainable. Therefore, success in sport must be measured in relation to specific contexts and circumstances [13].

Understanding how athletes perceive success in sport and how they set their standards and goals can provide insights into their motivation to continue engaging in sport [14]. Research has shown that even in competitive environments, such as NCAA college basketball in the USA, coaches often place significant importance on realizing each athlete’s potential, holistic development, and self-fulfillment. These aspects are considered essential parts of the definition of success in sport in these contexts [15]. Further research among coaches found that perceived success in sport includes the development of the players as a central component, followed by group performance, with victory only ranking in third place [16,17]. Emotional and personal growth was also identified as an additional component [18]. A study focusing on middle school athletes found that social orientations, such as team cohesion and peer relationships, were associated with pleasure and motivation as well as with perceived physical skills [19]. This suggests that defining success in social terms—such as the quality of relationships with teammates, the sense of belonging to a team, and the ability to work effectively with others—can help increase motivation in sport. Therefore, while individual achievements and ethical considerations are important, the social aspects of sport also play a crucial role in defining success.

Success in sport is influenced by various factors, including physical abilities (both innate and acquired), tactical, technical, and psychological aspects [20]. The athletes’ starting point, realistic expectations, and sports infrastructure also play a role in determining success. While winning the most Olympic medals may not be a feasible goal for all nations, even winning a single medal can be considered a significant achievement [20]. National success in sport is correlated with population size and economic wealth, and it can be defined relative to factors such as the sport’s prestige, financial investment, and participation opportunities [21]. Understanding and evaluating these parameters are crucial for comprehending the multidimensional nature of success in sport. Among these factors, mental strength, a psychological element, has been identified as a key differentiating factor between failure and success in sport [22,23]. It affects athletes’ performance in practice and competition, and understanding athletes’ perception of success and its impact on their attitude and behavior is important. In summary, success in sport is dynamic and influenced by individual circumstances. It can be measured through the achievement of specific goals, social recognition, and personal or group development [24]. Ultimately, success is a subjective attribute tied to an individual’s values and aspirations.

Several approaches have been utilized to investigate individual perceptions of success in sport. As far back as 1978, Nicholls [25] introduced two approaches to goal achievement within the framework of social cognitive theory, emphasizing the individual’s role in success and the inherent nature of success itself. Nicholls classified these approaches as “ego” and “task.” In the context of sport success, this dichotomy separates success factors into two categories: talent, and the dedication and effort invested in skill development. On the one hand, athletes may believe that investing their efforts in practicing over time is a factor in their success; on the other hand, they may see their born talent as a major factor distinguishing between themselves and other athletes [26]. According to Roberts et al. (1998), individuals who perceive success as a task approach view success as the point at which athletes demonstrate mastery of their performance abilities. This perspective encourages maximum investment and a forgiving attitude towards mistakes. It is a relative approach to success, allowing athletes to recognize their own potential for improvement without being adversely affected by perceived differences in talent compared to others [27]. In contrast, the ego perspective regards success in sport as relative to the victory achieved over other athletes in the same field [28] This approach attributes greater importance to the outcome of winning, rather than solely focusing on individual effort. Therefore, even if athletes exert maximum effort or execute the highest level of performance, they may not feel successful if they do not achieve victory over their competitors [14]. The emphasis on relative success in the ego perspective highlights the significance placed on comparative performance against others in determining success in sport.

Moreover, since persistence in sport is related to success as a motivation, success perceptions also contribute to the extent to which athletes continue to participate in sport over time [20]. Athletes’ success perceptions are influenced by several factors, among them coaches, the media, and their friends. Yet, these perceptions are also directly influenced by those of their parents [29]. When parents themselves believe that devoting effort to something is a major factor in success, their children will develop a tendency toward task perceptions of success in sport. This means they perceive success as a result of dedicated effort and skill development. On the other hand, if parents attribute success primarily to talent or external factors, their children are more likely to adopt ego perceptions of success [30]. Therefore, the beliefs and values that parents instill in their children regarding success significantly shape their perceptions and attitudes towards achieving success in sports. By emphasizing the importance of effort, parents can foster a mindset that promotes task-oriented success perceptions and encourages athletes to invest in their development and improvement.

Perceived career success encompasses both objective and subjective dimensions. Objective success is typically quantifiable, such as the income level, job conditions, and social prestige associated with certain professions [31,32]. On the other hand, subjective success pertains to an individual’s perspective and encompasses satisfaction, personal development, and a sense of fulfillment. In the realm of sports, similar to the career world, equal opportunities are lacking, and certain population groups face biases [6]. Consequently, international sports competitions may not serve as an accurate platform for objectively evaluating success, particularly for children who participate in sports or other niche areas with limited competition and audience exposure. Thus, it becomes crucial to examine the subjective nature of success. The existing research literature highlights individual and social distinctions in the perception of success. Duda et al. (1992) propose that cultural and gender differences also influence how children adopt goals and perceive success in sports. For instance, studies indicate that boys tend to prioritize ego-directed goals, while girls tend to lean towards task-oriented goals. Additionally, cultural factors such as individualism versus collectivism can shape the types of achievement goals children embrace [18].

Nonetheless, to the best of our knowledge, the discrepancies between children and parents in the context of perceived success in sport, both in general and particularly in children’s sport, are only marginal, and have not been directly examined until now. The current study aims to fill this research gap by focusing on the distinctions in perceived goals and success in sport between parents and children in the specific area of kayaking in Israel.

## 2. Methods

A qualitative research study was conducted to examine perceptions of success and goal setting, and to identify distinctions between parents and children. The study included a series of in-depth interviews with twenty participants: ten girls, aged 12–16, who train and compete in the sport of kayaking in Israel, and ten parents. We selected this demographic as it is often during this age range (12–16 years) that a shift from recreational to competitive sports is observed [33]. Consequently, we anticipated that elements such as competition and ambition, which are commonly linked with success, would be evident in the sporting journeys of these young female kayakers.

Moreover, at this age, the goals of engaging in sport become significant, including motivations and aspirations for success, as opposed to other personal and social incentives. The parents of the young athletes were also included in this study so as to generate a comparative perspective and to examine whether the views of the parents differ from those of the girls. When choosing participation, no consideration was given to the parents’ sporting history. Some participated in competitive sports as children or adults, while others did not. In the sport of kayaking, neither of the parents was a competitive athlete. Because there were no apparent distinctions between the interviewees’ responses, the parents’ individual backgrounds were not given any significance. All the girls who participated in the study train at the same kayaking club on the Sea of Galilee in northern Israel. ‘While we did not collect data on the participants’ income levels, we chose a single location for the study in an effort to minimize potential differences in demographic attributes. We acknowledge that this is an assumption and that variations in income level may still exist among the participants. Future research could benefit from directly measuring such demographic variables. The choice of a single location was designed to create homogeneity among the participants and to avoid major differences in demographic attributes, especially income level and geographic location, which are liable to influence the participants’ views.

In constructing the semi-structured interview, the researchers relied mainly on the Perception of Success Questionnaire [34]. Sample questions included: Why is success important to you (or your daughter)? What are her goals/your goals for your daughter’s participation? How do you see success, and on what factors is success dependent? Each of the young participants answered for herself and all the parents answered for their daughters. The interviews were conducted at the kayaking clubhouse before and after practices, as this was the most convenient time for both the young athletes and their parents. This timing also allowed us to conduct the interviews in a familiar environment, which we hoped would help the participants feel more comfortable and open in their responses. The interview stage lasted two months and entailed individual interviews that lasted 20 to 40 min each. The interviews were recorded with the approval of the interviewees and were subsequently transcribed. In some cases, topics that came up in the interviews were later clarified or completed by telephone or email. 

After that, each researcher separately conducted a thematic analysis of the interviews. The researchers adopted an inductive data analysis approach in which the themes emerged directly from the data without any pre-existing coding framework or predetermined categories. After the initial familiarization with the data, the researcher engages in open coding to capture the essence of specific concepts and ideas. The next phase included generating and refining the subthemes [35]. Among the sub-themes emerging from the analysis were achievement-oriented goals, enjoyment vs. achievements, success as a process, parents and direction toward enjoyment, and success as a subjective component. These sub-themes were narrowed down to three main themes: perceived success, goals vs. success, and perceptual gaps between parents and children. After separate analyses were conducted by each of the three researchers, they compared their results, and no significant differences emerged. 

No significant differences were found within each group of participants (girls and their parents). The age of the athletes and the number of years they participated in the sport did not yield any significant variance in the responses, and the interviewees’ responses were quite homogeneous.

The interviews were conducted in accordance with customary ethical standards approved by relevant Institutional Review Board. The parents gave written informed consent for their participation and that of their children. The research goals were explained to all the interviewees, who were told they could discontinue the interview at any time or choose not to answer particular questions. All participants cooperated fully. 

## 3. Discussion

### 3.1. Success in Kayaking

Even though kayaking is an individual sport, the practices take place in a group setting. Hence, while the group framework and the internal social aspects are important, the competitions and the performance during the practices are individual. In group sport, perceived individual success, which is an integral part of an athlete’s involvement in the sport, becomes intertwined with the overall success of the group [36]. Nevertheless, this sense of a shared destiny did not find expression in the responses of the participating athletes or their parents. It seems that their attitude toward success did not touch upon any external matters beyond sport. Their attitude toward the club was solely instrumental, and the club’s collective success by means of individual success was not reflected in the interviewees’ responses. Furthermore, even when the interviewees were asked about perceptions of success held by other athletes, their answers were not significantly different. The responses revealed that the interviewees projected their own ideas of success and sporting goals onto other members of the same group. The projection of attitudes on peers is relatively common in adolescents [37]. Thus, they believe that each athlete roughly shares the same attitude and aspirations.

Theories of perceived success in sport describe two distinct types. The first type is focused on task or mastery, whereas the second is focused on ego or victory over rivals. The implications of each of these types are described in the literature, yet for the most part, the division is not dichotomous. Rather, what emerges is a spectrum of subjective perceptions that, as can be seen in the findings of this study, are dependent upon time and on the process each individual athlete undergoes. Absolute expressions of task and ego as success in sport were found in all the interviews, whereas the expressions of each group were different, no significant tendency in a particular direction emerged. On the one hand, the participants pointed to self-improvement as a marker of success. A, for example, stated: “*We are competing in a branch of sport that is extremely precise. Based on our times, we know how good we are. We can also feel this but it’s important to know because there are all kinds of benchmarks along the way. All kinds of moments when you know you’ve improved. That today you are better than you were yesterday or a year ago.”* S added: *“It’s not important that I manage to outperform (name of a leading competitor in the group) but rather that it doesn’t break me, that I don’t drop out because I’m in second place. I think that what would be hardest would be to see that I am not outperforming my previous best times. That I’m not managing to improve in the ways that I and my coach would like.*” 

This aspect of self-improvement rather than improvement relative to others was even more prominent in the parents’ responses. G, for example, stated: “*We’re not here to win championships. Of course, that would be great and I think that would give my daughter a huge boost of motivation, but what is more important is for her to understand the association between hard work and results. For her to feel she was faster, not related to her place in any specific competition. Because there will always be someone who practices more or has more money for equipment and better coaches.*” In contrast, even in the same interview some of the participants, both parents and children, expressed the competitive view and the importance of ego in how success is perceived. A, for example, noted: “*Ultimately, it’s me against myself. It’s important for me to improve. However, frankly, it would be very nice to be the best. I think my parents would also be very proud if their daughter was the best in the club, that is, the best in the entire region. That’s not what I’m mainly looking for, but maybe to some extent I am.*”

The answers of all the interviewees in all the groups exhibited a high level of uniformity. Two interviewees attributed a significant degree of importance to victory and competitive achievements as markers of success. Nevertheless, most of the interviewees exhibited the full range of responses, with a tendency toward developing individual skills as the central component. This uniformity was also evident in the responses of the parents. While they tended more in the direction of task as a measure of success, they also included components of ego that can be perceived as success for them and for their children.

### 3.2. Participants’ Goals vs. Perceived Success

In most cases, success emerged as equivalent to achieving the goals the athletes set for themselves. Thus, in seeking to examine perceived success, we also explored the goals the interviewees set with respect to their participation in sport. The athletes themselves exhibited a significant tendency toward choosing sports achievements as a goal for participation. All the interviewees noted the competitive aspect, whether in terms of winning local competitions or of participating in the Olympic Games. R noted: “*I want to go as far as possible, to win all the competitions at all the levels.*” A added: “*I see myself competing abroad as well. That is the essence of the sport, to be the best*.” Yet, despite this strong position, this perspective was almost not present when the interviewees described what they believe is the greatest success in sport. M, for example, stated: “*In my view, success is to feel completely satisfied with what you do, to know that you are improving and managing to achieve things you were unable to do before.*” Some of the interviewees noted that for them success is fulfilling the goals each set for herself. Yet, when asked about these goals they did not refer again to high achievements and winning competitions but rather expressed their reservations and mentioned goals related to skills and self-improvement. These findings point to a distinction between their goals and their perceptions of success. The goals included athletic achievements, whereas success was not necessarily dependent upon this measure. Among other things, this discrepancy stems from a holistic perception of sport as providing education in values other than competitiveness [15,18]. 

This discrepancy has three possible explanations. First, as time goes by and participation in the sport continues, the participants’ goals may change. During practices and competitions, the athletes may become aware of their abilities vis à vis those of their competitors, and may thus begin to assess their realistic chances of achieving such levels when setting their goals. See, for example, the subjective view of success proposed by De Bosscher et al. [20]. Second, as these athletes gain experience in this sport and interact with the coaches, they may be exposed to other advantages of sport that are not dependent upon achievements, such as determination, persistence, self-confidence and body image [see, for example 18 regarding how coaches view success in sport]. A third explanation for the discrepancy between goals and the definition of success can be found in the athletes’ need to define success other than through objective measures to create a safety net as a means of coping with a lack of success. Only a small percentage of teenage athletes become professional, and of those, even fewer reach the top competitive levels. Hence, finding value in sport other than being victorious enables the athletes to justify their huge investment and to experience success through individual factors that are not absolute, such as pleasure, emotions, self-improvement, and more.

The findings from the interviews indicate that the young athletes formed their views on success as a tool for coping with the high goals they set for themselves. Nonetheless, it is important to note that the research participants are teenage girls who persisted in this sport and did not drop out. Those who dropped out over the years may have done so because they did not meet their goals, or were unable to see success in sport other than in terms of the sport’s competitive nature. Adolescents initially attribute particular importance to succeeding in their sport. Only after they become familiar with the sport are they able to see its additional advantages, which help them continue practicing. Changing these initial perspectives may lead more children and adolescents to engage in sport, help prevent disappointment, and thus keep them within the system for more time. Perceptions of success in sport can be shaped through guided intervention and by creating a particular sports climate [14]. Nevertheless, as discussed below, there is often a gap between the perceptions of the parents and those of the children.

### 3.3. The Gap between Young Athletes and Their Parents

Most of the studies in the literature demonstrate a correlation between the sports climate—particularly parental influence—and shaping young athletes’ perceptions of success in sport [14,17,38]. The findings of the current study to a large extent correspond to those of previous studies, apart from the specific context of setting initial goals for participation in sport. Indeed, the parents consistently mentioned pleasure as a primary and leading goal for their daughters’ participation in sport. While they did mention other motivations, such as boosting self-confidence, achievements, and personal fulfillment, the component of pleasure emerged in all the interviews with the parents. In contrast, the girls stressed competition alongside personal development, whereas pleasure was not mentioned at all. When asked directly about pleasure as a motivation for participating in sport or as a goal, the girls talked mainly about the social environment and intergroup relations with the other athletes, and not about pleasure derived directly from engaging in sport. N, for example, stated: “*Of course it’s fun. Otherwise I don’t think I’d stick at it. I like meeting with friends. I don’t think I really enjoy the practices themselves. Maybe afterwards I enjoy the feeling that I did something, but not as much as I enjoy my free time.”* S similarly commented: *“This doesn’t seem like a particularly enjoyable sport, like basketball, for example. It’s mainly more and more practices, and that in itself is really boring. My friends play basketball, and they pass, shoot, something different every time. It’s not simply paddling in a straight line.*” 

Their parents, in contrast, did refer to the pleasure derived from engaging in this sport. A stated: “This sport is the most fun. I remember myself going happily to the practices. It was an hour or more of pure joy. I do not know if my daughter feels the same. I think she does. She says it’s fun for her.” N referred directly to the pleasure he believed his daughter derived from the sport: *“First of all, she’s there because she enjoys it. She introduces herself that way and she talks about it with her friends that are not in this club. I’m pleased she enjoys it so much. From the outset that was important to me. I knew that being in the water and breaking the routine would be good for her.”* This discrepancy between the parents and their daughters stems from their differing perceptions of engaging in sports. The parents used the word “club” more frequently, indicating that they saw this as a leisure activity, and their descriptions referred mainly to their children’s educational activities. The young athletes primarily used the word “practices,” thus stressing the serious and more professional aspect of sports activity. From the outset, the parents referred to their daughters’ participation in this sport in terms of a holistic educational tool designed to provide a wide range of advantages for their children. Their daughters, in contrast, saw their involvement in terms of preparation to enter the world of competitive and professional sport. This perceptual difference also explains the girls’ focus on success in sport as the primary goal of their participation. Let us reiterate that the interviewees included only those who were still actively involved in kayaking over a long period of time, such that their attitude toward the sport derives from their investment. Their parents, in contrast, did not see their children’s participation similarly, and did not necessarily relate to their competitiveness on the same level. Unlike prior research [38], the results of this study show that parental perceptions are not necessarily passed on to their children, and children do not necessarily adopt similar views of success in sport. The strong influence of coaches along with the dynamic experience of the practices may also help shape children’s individual and independent perspectives.

## 4. Conclusions

The research literature in the field of success and goals in sport points to a dichotomy between improving skills and competitiveness. In the case examined in this study, the participants’ perceptions of success in sport range along the spectrum between these two components, with a tendency toward attributing more significance to the task factor and skill improvement. Nonetheless, this view of success is not in line with the goals of sports participation noted by the young athletes themselves. This discrepancy appears to stem from their view of the goal of sports participation in general and relative to the setting, and to themselves as competitive athletes. Their view stands in contrast to that of their parents, who see their daughters’ sports participation as a leisure activity and thus emphasize the component of pleasure. This component is exterritorial to the dichotomy between competitiveness and skill development, for it does not consider athletic achievements other than in terms of the athlete’s subjective experience. However, pleasure is indeed an essential component of persistence in sports activity [39]; the parents saw pleasure as an independent measure of success due to their attitude toward sport and its goals [40].

It should be noted that as a case study, the research herein has a limitation regarding the similarities between the participants’ socioeconomic backgrounds. This homogenizing of the interviewees could create a bias in the overall expectations, beliefs, and values of parents and children in reference to the factors that are being studied. This study represents an initial examination of perceived success among female athletes and their parents in a single branch of sport in Israel. It can serve as the basis for future research on social perspectives on sport, motivations for participation, and persistence over time.

## Data Availability

The data presented in this study are available on request from the corresponding author. The data are not publicly available due to privacy issues.
